# Parent concerns prior to an assessment of autism spectrum disorder: A systematic review

**DOI:** 10.1177/13623613241287573

**Published:** 2024-10-12

**Authors:** Dominique Solia, Loai Albarqouni, Paulina Stehlik, Antonia Conroy, Rae Thomas

**Affiliations:** 1 Bond University, Australia; 2 Griffith University, Australia; 3 Illume Development & Behaviour, Australia; 4 Tropical Australian Academic Health Centre, Australia

**Keywords:** assessment, autism spectrum disorder, child, development, diagnosis, parent concerns

## Abstract

Some parents experience concerns relating to their child’s development and seek clinical assessments for their child’s behaviour. We sought to understand the concerns that led parents to seek a general clinical assessment where some children were diagnosed with autism spectrum disorder. We conducted a systematic review to identify the types of parent concerns that drives parents to seek professional help for their child’s development. Database searching identified 7587 records, where 10 studies (9 cohorts) met our inclusion criteria. Parent reported concerns were categorised using the coding schemes of included studies described as autism spectrum disorder–specific (i.e. communication, social or stereotyped behaviour concerns) or non-autism spectrum disorder–specific concerns (i.e. behaviour/temperament, developmental, medical, sensory or motor concerns). The most reported parental concerns prior to an assessment were speech and language, social and behavioural. Concerns were also reported relating to parent understandings of an autism spectrum disorder diagnosis and the positive and negative implications surrounding the autism spectrum disorder diagnosis. Data were reported descriptively due to variations in study design and data analyses. Additional rigorous research on parent concerns prior to a diagnostic autism spectrum disorder assessment is required to capture what drives parents to seek professional help to support their child’s development.

**Prospero registration number:** CRD42022307436

**Lay abstract:**

When a parent has concerns about their child’s development, there is a lag between seeking and receiving health information. When waiting, parents may speculate about a possible diagnosis of autism spectrum disorder, but it is unclear what types of concerns might drive this speculation. To determine the types of concerns parents may have before their child is assessed, we conducted a systematic review that explored parent concerns before an autism spectrum disorder assessment. Our aim was to determine the types of concerns that might drive parents to seek medical help for their child’s development. Four online databases were searched and 10 articles reporting on 9 studies matched our inclusion criteria. In these cohorts, parents reported autism spectrum disorder–specific concerns (i.e. communication, social or stereotyped behaviour concerns) or non-autism spectrum disorder–specific concerns (i.e. behaviour/temperament, developmental, medical, sensory or motor concerns). Some parents also reported on their positive and negative thoughts of a potential autism spectrum disorder diagnosis and what the diagnosis would mean to them. The most reported parental concerns before an assessment were speech and language, social and behavioural. To understand the types of concerns parents have once they seek medical help, further research into how families speculated a possible autism spectrum disorder is recommended.

## Introduction

When children experience delays in meeting developmental milestones, parents may start to question whether the behaviours exhibited are problematic enough to warrant further investigation (Gentles et al., 2019). Parents begin the process of questioning whether their child may have autism spectrum disorder (ASD) by noticing that what seemed like minor developmental concerns might be more challenging and last longer than anticipated (Baghdadli et al., 2003; Gentles et al., 2019). This can be concerning for parents, as autistic individuals often experience and exhibit lifelong difficulties characterised by impaired social communication and interactions, and by restrictive, repetitive interests and behaviours (RRB) (American Psychiatric Association, 2013; World Health Organization, 2019).

Understanding what child behaviours trigger parents to explore reasons for the potential differences in their child’s development is key for paediatric healthcare workers to provide a parent-centred approach to support and educate parents (Johnson et al., 2020; Mintz, 2018). To understand early child behaviours that prompt parents’ concerns, studies have often retrospectively gathered this information. In these studies, parents of an autistic child are often asked to remember what their child’s behaviour was like prior to a diagnosis (Guinchat et al., 2012; Johnson et al., 2020; Sivberg, 2003; Waddington et al., 2023). However, retrospective parent-report has limitations. Parents’ recollection of concerns may be subjected to confirmation bias, as they may be more likely to only recall behaviours associated with the ASD diagnosis (i.e. social, communication, RRB) (Mitroulaki et al., 2022; Sacrey et al., 2015), rather than recall additional concerning behaviours that are not part of the ASD diagnosis (i.e. motor, medical, temperament) (Waddington et al., 2023). Detecting parental concerns prospectively avoids some biases associated with retrospective study designs and allows for tracking the emergence of concerning child behaviours prior to a possible ASD or other developmental diagnosis (Zwaigenbaum et al., 2007, 2009).

Parents and clinicians often differ in their language. Parents are not fluent in clinical terminology to communicate their concerns explicitly to clinicians, meaning their concerns may get lost in the translation process (Guinchat et al., 2012; Ryan & Salisbury, 2012). This can result in long delays between initial concerns raised, referral and possible diagnostic assessment (Brett et al., 2016; Mitroulaki et al., 2022; Ozonoff et al., 2009; Zuckerman et al., 2015) and can be a very stressful experience for parents (Crane et al., 2016). A better understanding of parent concerns and worries will enhance communication in initial consultations, allow parents to feel heard (Ryan & Salisbury, 2012) and improve clinical decisions (Mintz, 2018; Ozonoff et al., 2009). So, it is important we listen to families prior to a diagnostic outcome to better understand what concerns and worries drive parents to seek help (Gentles et al., 2019; Sivberg, 2003).

Therefore, we conducted a systematic review to synthesise parental concerns and worries before their child’s diagnostic assessment. Assessing concerns collected before parents are informed of the child’s diagnostic outcome will avoid confirmation bias and assist in understanding the common drivers in seeking a diagnostic assessment (Mitroulaki et al., 2022).

## Methods

### Design

We conducted a systematic review of studies that explored concerns and worries of parents prior to a diagnostic assessment. We prospectively registered the protocol for this review in PROSPERO (CRD42022307436) and followed the reporting standards of the Preferred Reporting Items for Systematic Reviews and Meta-Analyses (PRISMA) statement (Page et al., 2021). The protocol was followed with minor adjustments, which are reported in the ‘Methods’ section.

### Search strategy

To develop the search strategy, one author (D.S.) manually identified four parent concern studies that met the inclusion criteria for this systematic review (Jacobs et al., 2020a, 2020b; Pfeiffer et al., 2021; Wallisch et al., 2020). These studies were uploaded to an automated WordFrequency tool (Clark et al., 2020) which identified common words across the studies. These were used to formulate the search string which was reviewed by a senior research information specialist. We searched four electronic databases: PubMed, PsycInfo, Embase and CINAHL from inception until 28 February 2022 using a combination of free text and Medical Subject Headings (MeSH) terms about autism, concerns and diagnosis (see Supplementary File 1). We also conducted forward-backward citation searching of included studies. Studies were limited to those published in English with no publication date restrictions.

#### Eligibility criteria

##### Types of participants

We included studies of parents with children <18 years who had reported concerns about their child prior to undertaking a diagnostic assessment. We included studies regardless of the child’s diagnostic outcome, meaning all diagnostic outcomes were included: ASD diagnosis; non-ASD diagnosis; no diagnosis; and diagnostic outcome not determined.

##### Types of studies

We included studies with parent concerns collected prior to a diagnostic assessment via open-ended survey questions, clinical notes taken during medical consultations, and/or interviews. Concerns must have been recorded via open-ended questions as we wanted direct information from parents rather than imposing preconceived categories by checklists. We excluded studies with polar questions (including those with lists of behaviours for parents to tick) to avoid bias in parent responses. We also excluded studies with a time gap of greater than 12 months between the recording of parent concerns and a diagnostic assessment or if concerns were recorded retrospectively.

##### Types of outcome measures

Our primary outcome was the concerns reported by parents considering a diagnostic assessment of ASD for their child. We included studies where parent concerns were reported qualitatively or quantitatively (where content coding was conducted by the authors of the original studies).

#### Selection of studies

##### Study selection and screening

Three reviewers (D.S., P.S., A.C.) screened 15% of title and abstracts in duplicate against the inclusion and exclusion criteria, and interrater reliability was established at 0.99. One reviewer (D.S.) continued screening remaining studies. Queries or disagreements were resolved through discussion with a third party (L.A.). The same process was followed for full-text review.

##### Data extraction

One reviewer (D.S.) extracted data while a second (P.S.) checked a random sample of 50% of included studies for accuracy. Information extracted included study characteristics (i.e. location, study design, recruitment, interventions, referral, inclusion and exclusion criteria, parent cohort size and education, socio-economic status, children cohort, children gender and ethnicity, age of first concern, age at assessment, diagnostic outcome), data collection method, coding technique and parent concerns (i.e. ASD-specific and non-ASD-specific concerns). Parental concerns were extracted as reported in the included studies and sorted into the two categories, ASD-specific and non-ASD-specific concerns, an approach originated from Ozonoff et al. (2009) and its adaptations (Donohue et al., 2019; Richards et al., 2016). ASD-specific concerns are behaviours assessed against ASD diagnostic criteria (American Psychiatric Association, 2013; World Health Organization, 2019) and were coded as speech/language/communication, social and stereotyped behaviours. Non-ASD-specific concerns are categorised by the selected coding criteria as behaviour and temperament, general development, sensory, motor, and medical and regulatory are general concerns about the child’s development (Donohue et al., 2019; Ozonoff et al., 2009; Richards et al., 2016). We note that sensory differences are a diagnostic criterion for ASD (American Psychiatric Association, 2013; World Health Organization, 2019); however, as the coding criteria is regularly used in the literature and many of our included studies adhered to this criteria, we have kept it under non-ASD-specific concerns for consistency.

##### Risk of bias

The Mixed Methods Appraisal Tool (MMAT) (Hong, Fàbregues, et al., 2018; Hong, Gonzalez-Reyes & Pluye, 2018) was used to assess the risk of bias in included studies. The MMAT tool is designed to appraise mixed method systematic reviews that include qualitative, quantitative and mixed method studies using quality criteria (see Supplementary File 2) (Hong, Fàbregues, et al., 2018). Two reviewers (D.S. and A.C.) appraised 50% of included studies independently to check for accuracy. Interrater reliability was established at 0.83 and one reviewer (D.S.) continued to screen the remaining studies. Any uncertainties were subsequently resolved via a third reviewer (L.A.).

##### Data analysis/synthesis

Due to disparate study designs and data collection measures, data were synthesised narratively. Studies that used content analyses to code parent concerns against ASD diagnostic criteria were extracted and reported verbatim into ASD-specific and non-ASD-specific categories (Ozonoff et al., 2009). These categories were used, as parents may have difficulty in recognising and communicating concerning behaviours during a child’s early development (Guinchat et al., 2012), and this method relies on researcher interpretation of parent responses to place the concern into categories (Pfeiffer et al., 2021). Studies that reported on themes derived from parent worries around a diagnosis were extracted as is, to ensure authors abstracted themes were retained.

##### Community involvement

A developmental and behaviour consultant (A.C.) who works with autistic children was involved in refining the research question, screening the articles for inclusion, data extraction and interpretation of the findings.

## Results

We identified 6835 records through database searching and 752 through forward-backward citation searching of included articles. We excluded 4576 records after title and abstract screening and reviewed 71 studies for inclusion. We included 10 studies (9 cohorts) (Supplementary File 3).

### Study characteristics

Seven cohorts were conducted in the United States (Azad et al., 2022; Coffield et al., 2020; Coonrod & Stone, 2004; Herlihy et al., 2015; Hess & Landa, 2012; Richards et al., 2016; Wallisch et al., 2020), one in Belgium (Jacobs et al., 2020a, 2020b) and another in Malaysia (Jayanath & Ozonoff, 2020). Eight studies reported on behaviour concerns relating to ASD diagnostic criteria and one study reported on parent motivation to request a diagnostic assessment (Jacobs et al., 2020a, 2020b). Eight studies collected parental concerns prospectively when parents sought medical advice (Azad et al., 2022; Coffield et al., 2020; Coonrod & Stone, 2004; Herlihy et al., 2015; Jacobs et al., 2020a, 2020b; Jayanath & Ozonoff, 2020; Richards et al., 2016; Wallisch et al., 2020), while one prospective longitudinal study included only participants who had an older autistic sibling (Hess & Landa, 2012).

Parental concerns of child behaviour were recorded and diagnostic assessments were conducted at ASD speciality clinics (Azad et al., 2022), university clinics and diagnostic centres (Coffield et al., 2020; Coonrod & Stone, 2004; Herlihy et al., 2015; Hess & Landa, 2012; Richards et al., 2016; Wallisch et al., 2020), paediatric clinics (Jacobs et al., 2020a, 2020b; Jayanath & Ozonoff, 2020) and a child psychiatric clinic (Jacobs et al., 2020a, 2020b). Most studies gathered concerns by written open-ended questionnaires (i.e. intake surveys, paperwork and/or screening tools) (Azad et al., 2022; Coffield et al., 2020; Coonrod & Stone, 2004; Hess & Landa, 2012; Richards et al., 2016; Wallisch et al., 2020). One study used semi-structured parent interviews (Coffield et al., 2020) and one collected data from electronic medical records from doctor’s consultations (Jayanath & Ozonoff, 2020). Participants in one prospective longitudinal study were enrolled as they had an older autistic sibling.

One study conducted in-depth phenomenological interviews of one or both parents to investigate how parents understand and experience a possible ASD diagnosis and what parents think and feel when they ask for a diagnostic ASD assessment. These were collected in a series of semi-structured interviews and were used flexibly for participants to elaborate freely (Jacobs et al., 2020a, 2020b).

### Participant characteristics

A total of 1258 parents reported concerns for 2391 children (male 76%) of whom 1725 (72%) received an ASD diagnosis (Table 1). Three studies did not report on the sampled number of parents (Azad et al., 2022; Jayanath & Ozonoff, 2020; Wallisch et al., 2020). Four cohort studies included all participants regardless of children’s diagnostic outcomes (Coffield et al., 2020; Hess & Landa, 2012; Jacobs et al., 2020a, 2020b; Richards et al., 2016) and five cohorts limited to reporting on participants depending on children’s diagnostic outcomes (Azad et al., 2022; Coonrod & Stone, 2004; Herlihy et al., 2015; Jayanath & Ozonoff, 2020; Wallisch et al., 2020). Participants’ socio-economic status was reported in one study (Hess & Landa, 2012) and one defined their cohort as medically underserved due to participants’ geographic locations (Coffield et al., 2020). Three cohorts were part of longitudinal studies (Herlihy et al., 2015; Hess & Landa, 2012; Jacobs et al., 2020a, 2020b), with one validating the Modified Checklist for Autism in Toddlers–Revised (M-CHAT-R) screening tool (Herlihy et al., 2015), and another followed younger siblings of autistic children (Hess & Landa, 2012). Additional study characteristics are reported in Supplementary File 4.

**Table 1.** Study characteristics.

Studies	Recruitment process	Diagnostic centre	Instruments administered before parent concerns collected	Population	Children age in years M (SD) [range]	Children Ethnicity (%)	Risk assessment	ASD diagnosis %
Azad et al. (2022), United States of America	Referred by: • Paediatricians (41%). • Specialists (49%). • Other (10%).	University affiliated, ASD Specialty Clinic.	Questionnaires: • Clinic-developed intake questionnaire. Background and history form.	Parents: • Not reported. Children (*n* = 489): Male (79%).	5.4 (3.4) [1.2–15.0]	White (48%) Asian (10%) Black/African American (23%) Hispanic (6%) Multiracial or Other (13%)	NR	100%
Coffield et al. (2020), United States of America	Recruited by: • Participants from the developmental evaluation clinic were invited to participate. Referred by: Participants from the Federally qualified health centres were referred by staff.	Developmental evaluation clinic. Federally qualified health centres.	Prior Screening: • M-CHAT-R. • The Social Communication Questionnaire. • Developmental Check-In. Interview: • Structured parent interview. Gathered health information.	Parents (*n* = 204). Children (*n* = 288): Male (74%).	3.0 [2.0–5.0]	Black (20%) White (36%) Other (20%) Multi-Race (1%) No Response (22%) Hispanic (54.86%)	NR	73%
Coonrod and Stone (2004), United States of America	Recruited by: Regional diagnostic centre where parents gave informed consent to the research.	Regional diagnostic centre.	Questionnaires: Early Concerns Questionnaire.	Parents (*n* = 45): • Mothers (91%). • Fathers (7%). • Caregiver (2%). Children (*n* = 44): Male (82%)	2.6(0.26) [2.0–3.0]	Caucasian (82%) African American (14%) Other ethnicities (4%)	NR	50%
Herlihy et al. (2015), United States of America	Selected from: A larger ongoing study designed to validate M-CHAT-R.	University of Connecticut.	Prior Screening: • M-CHAT-R. Questionnaires: History Form: 156-item questionnaire with multiple-choice and open-ended questions.	Parents (*n* = 71): • Mothers (89%). • Fathers (11%). Children (*n* = 69): Male (78%).	2.14	White (70%) Hispanic/Latino (12%) African American (7%) Biracial (4%) Unknown (7%)	High-risk and low-risk children	100%
Hess and Landa (2012), United States of America	Recruited from: A federally funded research project focusing on early markers for ASD.	Kennedy Krieger Institute or Spaulding Rehabilitation Centre.	Three short questionnaires: • Intake form where parents were asked to list concerns. • The Communication and Symbolic Behaviour Scale Developmental Profile-Caregiver Questionnaire. The Sensory Profile.	Parents (*n* = 63). Children (*n* = 89): Male (53%).	3.0	Caucasian (87%) African American (1%) Latino (1%) Multiracial (9%) Unspecified (2%)	High-risk children	27%
Jacobs et al. (2020a), Belgium Jacobs et al. (2020b), Belgium	Referred by: Clinicians who conducted the child’s assessment.	Two specialised centres: A child psychiatric and a paediatric centre.	Data Collection: In-depth interviews with parents before and after their diagnostic assessment.	Parents (*n* = 17). Children (*n* = 11): Male (100%).	3.65 (1.42) [1.9–6.3]	Flemish (100%)	High risk and low risk	100%
Jayanath and Ozonoff (2020), Malaysia	Selected from: Medical records of all patients (aged 1–18) at time of confirmed ASD diagnosis.	Developmental Paediatrics Clinic of University of Malaya Medical Centre.	Data Collection: Electronic Medical Records	Parents: • Not reported. Children (*n* = 366): Male (82%).	[1.0–18.0]	NR	High-risk and low-risk children	100%
Richards et al. (2016), United States of America	Selected from: Two larger ongoing studies designed to validate M-CHAT-R.	University of Connecticut.	Prior Screening: • M-CHAT-R. Questionnaires: Detailed history forms.	Parents (*n* = 858): • Mothers (52%). • Fathers (48%). Children (*n* = 532): Male (70%).	2.14 (0.39) [1.3–3.6]	Caucasian (72%) African American (32%) Hispanic/Latino (21%) Other (14%)	Low-risk children	52%
Wallisch et al. (2020), United States of America	Selected from: Medical records of children (36–72 month) with confirmed ASD diagnosis.	University-based child diagnostic centre.	Questionnaire: Intake paperwork for developmental history and behavioural information.	Parents: • Not reported. Children (*n* = 503): Male (81%).	4.5 [3.0–6.0]	White (75%) Black (13%) Two or more (9%) Asian (3%) American Indian/Alaskan Native (2%) Hispanic (12%)	NR	52%

ASD: autism spectrum disorder; M-CHAT-R: Modified Checklist for Autism in Toddlers–Revised.

### Main findings

Eight studies reported parent concerns of child behaviour and adopted content coding in line with diagnostic criteria or tools used to diagnose ASD (American Psychiatric Association, 2013; Lord, Luyster, et al., 2012; Lord, Rutter, et al., 2012; World Health Organization, 2019). Studies either used codebooks designed by the researchers (Azad et al., 2022; Coonrod & Stone, 2004; Herlihy et al., 2015; Hess & Landa, 2012), or used a coding scheme developed by Ozonoff et al. (2009) or its adaptations (Coffield et al., 2020; Jayanath & Ozonoff, 2020; Richards et al., 2016; Wallisch et al., 2020). The codebooks and coding scheme categorised developmental behaviours commonly observed in autistic children. Parental concerns were categorised into ASD-specific concerns (i.e. speech/language/communication, social and stereotyped behaviours) and non-ASD-specific concerns (i.e. behaviour and temperament, general development, sensory, motor, and medical and regulatory). Table 2 presents the code descriptions and matched examples of parental concerns.

**Table 2.** Extracted definitions.

Category	Description	Example
***ASD-specific concerns***		
Speech, language, and communication	Intentional communication, both receptive and expressive.	‘Won’t try to repeat words’ (Wallisch et al., 2020). ‘Behind on speech, would like for her to be able to express herself better’ (Coffield et al., 2020).
Social	Social engagement with other people, social or emotional reciprocity and social attention including eye contact.	‘My son doesn’t have friends his age’ (Wallisch et al., 2020). ‘Stares at other children and adults he is not familiar with. Will not react when they talk to him or try to play with him’ (Coffield et al., 2020).
Stereotyped behaviours	Rigid, repetitive, or odd behaviour such as object use.	‘Lines up his cars and trains and is fixated on them being in a straight line’ (Coffield et al., 2020). ‘He likes to spin in circles’ (Richards et al., 2016).
***Non-ASD-specific concerns***		
Behaviour and temperament	Ability to express, understand or regulate emotions and capabilities in internalising and externalising behaviours.	‘Has a very difficult time understanding and processing his emotions’ (Azad et al., 2022). ‘Aggressive, very easily frustrated and gets upset when he is not in control of the situation he is in or things happening around him’ (Coffield et al., 2020).
General development	Reaching developmental milestones, as well as cognitive and self-help concerns.	‘Developmentally behind others his age’ (Azad et al., 2022). ‘Does not do what other children [her] age do. Does not self-eat, does not drink the bottle by herself’ (Coffield et al., 2020).
Medical and regulatory	General health problems including specific medical issue, condition or psychological functions.	‘Doesn’t sleep for more than 2 hours straight at night’ (Coffield et al., 2020). ‘Is he having seizures?’ (Wallisch et al., 2020).
Sensory	Sensory interests or sensory aversions.	‘He smells everything’ (Wallisch et al., 2020). ‘Sensitive to light and noise’ (Azad et al., 2022).
Motor	Delays in meeting motor milestone or clumsiness.	‘Poorly developed fine motor skills and gross motor skills’ (Azad et al., 2022). ‘Coordination needs work. Falls a lot and runs into things’ (Coffield et al., 2020).

ASD-specific and non-ASD-specific concern categories and their definitions as described by included studies.

### ASD-specific concerns

**Figure 1.**
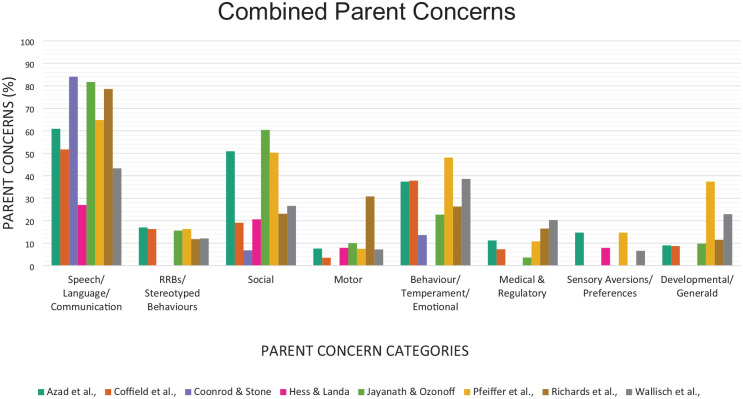
Parent concerns reported. All parent concerns reported were included in analysis in Azad et al. (2022), Coffield et al. (2020), Coonrod and Stone (2004), Herlihy et al. (2015), Hess and Landa (2012) and Richards et al. (2016). Top five parent concerns reported and analysed in Jayanath and Ozonoff (2020), and top three parent concerns analysed and reported in Wallisch et al. (2020). Additional information reported in supplementary file 2.

Irrespective of whether the assessment clinic was specialised or general, *Speech, language, and communication concerns* including expressive and receptive communication concerns was consistently the most common reported concern across all studies ranging between 27% and 84% of reported concerns (Figure 1 and Supplementary File 5). *Social concerns* relating to a child’s social and play skills were also commonly reported; however, this differed depending on the cohort’s age. Studies of older children reported a higher number of social concerns with 51% of parents in Azad et al. (2022) and 60% of parents in Jayanath and Ozonoff (2020) reported concerns, compared to studies of younger children with 7% to 27% of parents reporting social concerns (Coffield et al., 2020; Coonrod & Stone, 2004; Herlihy et al., 2015; Hess & Landa, 2012; Richards et al., 2016; Wallisch et al., 2020).

### Non-ASD-specific concerns

*Behaviour and temperament concerns* (internalising and externalising behaviours) were commonly raised by parents across all studies and ranged from 6% to 17% (Supplementary File 5), with age of children not impacting these results. Concerns relating to developmental milestones and cognitive and self-help concerns (*General development*) were raised in five studies (ranging from 9% to 23%) and in both ASD and general clinics, with age of children not impacting results (Azad et al., 2022; Coffield et al., 2020; Jayanath & Ozonoff, 2020; Richards et al., 2016; Wallisch et al., 2020). *Medical/regulatory* concerns were raised in five studies where concerns were raised between 4% and 20% of parents. A child’s sensory interests or sensory aversions were coded as *sensory concerns* with three studies reporting this concern (Azad et al., 2022; Hess & Landa, 2012; Wallisch et al., 2020). Overall, the least frequently expressed concern was for fine and/or gross motor or body movements (*Motor concerns*). However, there was wide variation, with one study reporting 30% (Richards et al., 2016) expressed concerns while the remaining five studies had a range of 3% and 8% (Coffield et al., 2020; Hess & Landa, 2012; Jayanath & Ozonoff, 2020; Wallisch et al., 2020).

### Parent concerns and diagnosis relationship

Parents of autistic children (i.e. those children eventually diagnosed with ASD) were more likely to report ASD-specific concerns in the majority of studies (Coffield et al., 2020; Coonrod & Stone, 2004; Hess & Landa, 2012; Richards et al., 2016; Wallisch et al., 2020), whereas parents of children not diagnosed with ASD were more likely to report medical and/or behavioural concerns (Hess & Landa, 2012; Richards et al., 2016; Wallisch et al., 2020). Wallisch et al. (2020) reported a relationship with the types of parental concerns reported and the child’s diagnostic outcome. Their results indicate that compared with autistic children, children who were diagnosed with additional or different diagnoses had significantly or likely more *behaviour concerns, medical and regulatory concerns*, and fewer *speech, language, and communication* and *social concerns*. The results also showed that in comparison to autistic children, parents of children diagnosed with speech and language disorders were more likely to report *speech, language and communication concerns*.

### ASD diagnosis views and experiences

One cohort study (Jacobs et al., 2020a, 2020b) reported on experiences of the interviewed parents prior to the ASD diagnostic assessment, and specifically the various implications parents expected of the child’s possible ASD diagnosis. Jacobs et al. (2020a) applied interpretative phenomenological analysis (Smith et al., 2009) to the transcripts from semi-structured interviews with parents prior to a clinical diagnosis to investigate parental concerns. Four themes were derived from the interviews: parents and professionals observing worrisome behaviours; feelings about the possible ASD diagnosis and consequences and ASD getting mentioned; parents having ambiguous feelings about the consequences of a possible ASD diagnosis; and involvement of professionals addressing the possible need for a diagnostic ASD assessment (Jacobs et al., 2020a). The theme of having ambiguous feelings about the possibility of an ASD diagnosis had both positive and negative connotations and consequences. Negative implications included parents having an understanding ASD is a condition for life and fear of social reactions of stigmatising and stereotyping the child. Positive implications consisted of treatment-related and psycho-relational implications. Overall, parents wished to understand what was going on with their child and hoped for an exculpatory effect of the ASD diagnosis (Jacobs et al., 2020b).

### Risk of bias assessment

Overall, the risk of bias in the included studies appears low. All studies met the first two screening criteria for MMAT which assessed if there were clear research methods and if the data collected addressed these questions. All quantitative studies also met the MMAT criterion that participants be representative of the study’s target population; however, we applied a stricter criterion for this question and based our assessment on whether participants were representatives of the general population who undergo a diagnostic assessment. When this was applied, only half of the quantitative studies met this criterion. The qualitative study met all quality appraisal criteria (Jacobs et al., 2020a, 2020b). In the two mixed method studies, one met all criteria except for recruiting only autistic participants (Azad et al., 2022) and one met all criteria except there was no justification of using a mixed-methods study design and authors were unclear on their reporting on divergencies (Coffield et al., 2020). The remaining six quantitative, descriptive studies met all MMAT criteria except where participants were recruited retrospectively dependent on a receiving positive diagnostic outcome (Coonrod & Stone, 2004; Jayanath & Ozonoff, 2020; Wallisch et al., 2020) or where reporting was unclear on how many participants were excluded and how this impacted results (Coonrod & Stone, 2004; Herlihy et al., 2015; Hess & Landa, 2012; Jayanath & Ozonoff, 2020; Richards et al., 2016; Wallisch et al., 2020). See Supplementary File 6 for MMAT appraisal results.

## Discussion

The present study aimed to synthesise the evidence of common concerns expressed by parents prior to a child’s clinical assessment with a particular focus on children suspected to have ASD. Parent concerns reported across majority of studies were analysed and coded to align with ASD diagnostic criteria (*Speech, language and communication concerns, social concerns*, and *stereotyped concerns*) and non-ASD-specific concerns (*Behaviour and temperament concerns, general development concerns, medical and regulatory concerns, sensory concerns* and *motor concerns*) (Donohue et al., 2019; Ozonoff et al., 2009; Richards et al., 2016). Parent worries and concerns around a possible diagnosis was reported in one study (Jacobs et al., 2020a). To ensure authors meanings were retained, we extracted the derived themes as reported by Jacobs et al. (2020a).

It is worth noting that most participants were not representative of the general population of children referred for a diagnostic ASD assessment. The prevalence of a diagnostic ASD outcome in children who are referred for an assessment is between 61% and 70% (Bernie et al., 2020; Lo et al., 2017; Monteiro et al., 2015). However, the participants in our study had a diagnostic rate between 27% and 100% (Table 1), with majority of studies only reporting on participants with positive diagnostic outcomes (Azad et al., 2022; Coonrod & Stone, 2004; Herlihy et al., 2015; Hess & Landa, 2012; Jayanath & Ozonoff, 2020; Wallisch et al., 2020). Due to participant sampling, we were unable to determine parent’s spontaneous cause for concern or detect their motives for seeking professional advice (Gentles et al., 2019; Guinchat et al., 2012).

The frequency of parent concerns reported across studies may have impacted our results due to the various ways data was collected (Chawarska et al., 2007). Three studies screened participants for ASD prior to collection of concerns and before children were assessed (Coffield et al., 2020; Herlihy et al., 2015; Richards et al., 2016), and two of these studies informed parents of the screening results (Herlihy et al., 2015; Richards et al., 2016). This could have potentially made parents more aware of their child’s potential developmental delays, and thus, their responses could have reflected preconceived knowledge about their child’s development (Pfeiffer et al., 2021). Furthermore, parents in Richards et al. (2016) were prompted to focus on specific developmental concerns such as walking, speaking, playing or behaving which may have led to the high number of motor concerns reported (Concerns reported: 31%) (Richards et al., 2016), compared to all other studies (range: 3%–8%) (Azad et al., 2022; Coffield et al., 2020; Hess & Landa, 2012; Jayanath & Ozonoff, 2020; Wallisch et al., 2020). The collection methods used in these studies (Coffield et al., 2020; Herlihy et al., 2015; Richards et al., 2016) likely imposed preconceived ideas or professional terminology into parent responses (Pfeiffer et al., 2021).

No studies reported on parent ASD understandings prior to seeking professional help and many studies did not report on the services accessed prior to collecting parent concerns. Parent knowledge and awareness of what an ASD diagnosis entails, and their experiences of services accessed prior to a diagnostic ASD assessment can potentially impact the type of concerns acquired (Hrdlicka et al., 2016; Rosenbrock et al., 2021). This was demonstrated in Richards et al. (2016), where authors reported that the types of therapists and specialists a child saw were associated with the parent’s specific concerns. This scarcity of reported data may have contributed to the heterogeneity of results.

A range of cultures were included in the identified studies; however, only two conducted in the United States reported on parents’ first language and the impacts it may have had on parent concerns or perceptions (Azad et al., 2022; Coffield et al., 2020). Parent cultural conceptualisations and first languages can contribute to discrepancies in concerns reported at the timing of a diagnosis (Weitlauf et al., 2024). Parents may not have heard of ASD or know its symptoms or they may be reluctant to mention concerns due to cultural factors (Zuckerman et al., 2014). Coffield et al. (2020) and Azad et al. (2022) found that the number of parental concerns reported were dependent on either the parent’s primary language or by parent-reported race. Parents who spoke English as their primary language in Coffield et al. (2020) had significantly more concerns overall and more ASD-specific concerns than Spanish-speaking parents. Azad et al. (2022) reported on average that parents of Asian, Black/African American, and multiracial or other children had fewer number of concerns reported compared to those of White children. It was also reported that the length of parent concern responses differed by race and was longest in White parents, followed by Multiracial or other, Asian, Hispanic and Black/African American parents (Azad et al., 2022). As clinicians rely on parent concern reports to trigger referrals for assessment, and if multicultural or non-English-speaking parents under-report concerns specific to ASD or other developmental disorders, it may influence clinicians to delay further investigation (Donohue et al., 2019; Zuckerman et al., 2015).

As the number of children diagnosed with ASD increases, and parents become more aware of behaviours suggestive of diagnosis, it is important we examine the parental concerns, and the language parents may use to describe those concerns. In doing so, clinicians will be able to more effectively elicit parental concerns raised in consultations and thus improve clinical decisions (Mitroulaki et al., 2022; Ozonoff et al., 2009). The results from our study highlight that this is an important area of research but more rigour in study design that eliminates confirmation bias could provide a clearer understanding of parental worries. As all participants in our included studies were either referred to a diagnostic provider or were part of a longitudinal study, we were unable to detect parents’ spontaneous cause for concern or review concerns from parents who did not receive a referral.

### Strengths, limitations and future recommendations

To our knowledge, this is the first systematic review that examines parental concerns when presenting to a clinician’s office for a diagnostic assessment. The use of a defined inclusion-exclusion criteria, rigorous search from four databases with no additional articles found through forward-backward citation searching and assessing the quality of studies with a validated tool are considered strengths of this review. While partial duplication of screening, data extraction and risk of bias assessments yielded high interrater reliability, implementing full duplication in future reviews would further strengthen the methodological rigour and minimise potential errors.

The findings from our review should be interpreted with caution due to several limitations. First, to ensure results can be applied to patients in general primary care settings, future studies should consider how cohorts are recruited to capture parents’ causes for concern and their motives for seeking professional advice. Second, limited information was reported on therapies and services children had accessed prior to collection of parent concerns. As this paucity of information poses a risk of bias, future studies need to consider collecting the exposures to therapies and services families have previously had as this may influence the type and the number of concerns reported. Third, studies reported limited information on participants’ ethnicity, language, education, socio-economic status and health literacy and the impact these confounders have on the reported concerns. It is important that future research addresses these areas. Fourth, the location of studies was limited to those conducted in mainly the United States and one in both Malaysia and Belgium. To have an understanding how concerns differ across countries and cultures, it is essential that future research occurs outside of the identified places and includes more diverse ethnic and cultural populations. In addition, as this review only identified one qualitative study on concerns parents experience in the waiting period prior to a diagnostic ASD assessment (Jacobs et al., 2020a, 2020b), it is suggested that future studies exploring parental concerns prior to a diagnostic ASD assessment consider exploring the underlying worries families may experience outside of the child’s atypical behaviours. Better understanding of parents’ requests for help will help clinicians to address parents’ needs more effectively. Further and more rigorous research addressing the areas of limitations identified is needed to understand what drives parents to explore an ASD assessment and their underlying worries.

## Conclusion

This systematic review synthesises parental concerns collected prior to their child’s autistic and general assessments from qualitative, quantitative and mixed method studies. The results imply that parents have a range of concerns that are both ASD-specific and non-ASD-specific. Due to the types of studies included in this review, we were unable to determine parents’ spontaneous cause for concern to seek professional help. Further research on concerns parents develop before seeking medical help is required to help collectively understand what drives parents to seek a diagnostic assessment and how this may enhance clinician–parent communication.

## Supplemental Material

sj-docx-1-aut-10.1177_13623613241287573 – Supplemental material for Parent concerns prior to an assessment of autism spectrum disorder: A systematic review Supplemental material, sj-docx-1-aut-10.1177_13623613241287573 for Parent concerns prior to an assessment of autism spectrum disorder: A systematic review by Dominique Solia, Loai Albarqouni, Paulina Stehlik, Antonia Conroy and Rae Thomas in Autism
