# Supplementary material for: Parent concerns prior to an assessment of autism spectrum disorder: A systematic review
Source: Autism. 2024 Oct 12;29(4):838–49. doi: 10.1177/13623613241287573 (PMC11967076; doi:10.1177/13623613241287573)
Supplement: sj-docx-1-aut-10.1177_13623613241287573 – Supplemental material for Parent concerns prior to an assessment of autism spectrum disorder: A systematic review [file sj-docx-1-aut-10.1177_13623613241287573.docx]

**Supplementary File 1. Search String**

| **Database** | **Search String** |
| --- | --- |
| **PubMed** | ("parents"[Mesh] OR "caregivers"[Mesh] OR parent[tiab] OR parents[tiab] OR parental[tiab] OR mother[tiab] OR mothers[tiab] OR father[tiab] OR fathers[tiab] OR carer[tiab] OR carers[tiab] OR caregiver[tiab] or caregivers[tiab])  AND  ("Emotions"[Mesh] OR concern[tiab] OR concerns[tiab] OR worry[tiab] OR worries[tiab] OR wellbeing[tiab] OR well-being[tiab] OR "well being"[tiab] OR emotion[tiab] OR emotions[tiab] OR emotional[tiab])  AND  ("child development disorders, pervasive"[Mesh] OR ASD[tiab] OR autism[tiab] OR "autism spectrum disorder"[tiab] OR autistic[tiab] OR "pervasive developmental disorder"[tiab] OR PDD- NOS[tiab] OR asperger[tiab] OR aspergers[tiab])  AND  ("diagnosis"[Mesh] OR "risk"[Mesh] OR Diagnosis[tiab] OR diagnostic[tiab] OR diagnosed[tiab] OR screening[tiab] OR risk[tiab] OR risks[tiab]) |
| **PsycInfo** | (exp parents/ OR exp caregivers/ OR parent.ti,ab. OR parents.ti,ab. OR parental.ti,ab. OR mother.ti,ab. OR mothers.ti,ab. OR father.ti,ab. OR fathers.ti,ab. OR carer.ti,ab. OR carers.ti,ab. OR caregiver.ti,ab. OR caregivers.ti,ab.)  AND  (exp Emotions/ OR concern.ti,ab. OR concerns.ti,ab. OR worry.ti,ab. OR worries.ti,ab. OR wellbeing.ti,ab. OR well-being.ti,ab. OR "well being".ti,ab. OR emotion.ti,ab. OR emotions.ti,ab. OR emotional.ti,ab.)  AND  (exp autism spectrum disorders/ OR ASD.ti,ab. OR autism.ti,ab. OR "autism spectrum disorder".ti,ab. OR autistic.ti,ab. OR "pervasive developmental disorder".ti,ab. OR "PDD- NOS".ti,ab. OR asperger.ti,ab. OR aspergers.ti,ab.)  AND  (exp diagnosis/ OR exp risk factors/ OR Diagnosis.ti,ab. OR diagnostic.ti,ab. OR diagnosed.ti,ab. OR screening.ti,ab. OR risk.ti,ab. OR risks.ti,ab.) |
| **Embase** | (parent/exp OR caregiver/exp OR parent:ti,ab OR parents:ti,ab OR parental:ti,ab OR mother:ti,ab OR mothers:ti,ab OR father:ti,ab OR fathers:ti,ab OR carer:ti,ab OR carers:ti,ab OR caregiver:ti,ab OR caregivers:ti,ab)  AND  (Emotion/exp OR concern:ti,ab OR concerns:ti,ab OR worry:ti,ab OR worries:ti,ab OR wellbeing:ti,ab OR well-being:ti,ab OR "well being":ti,ab OR emotion:ti,ab OR emotions:ti,ab OR emotional:ti,ab)  AND  (autism/exp OR ASD:ti,ab OR autism:ti,ab OR "autism spectrum disorder":ti,ab OR autistic:ti,ab OR "pervasive developmental disorder":ti,ab OR "PDD- NOS":ti,ab OR asperger:ti,ab OR aspergers:ti,ab)  AND  (diagnosis/exp OR risk/exp OR Diagnosis:ti,ab OR diagnostic:ti,ab OR diagnosed:ti,ab OR screening:ti,ab OR risk:ti,ab OR risks:ti,ab) |
| **CINAHL** | ((MH parents+) OR (MH caregivers+) OR (TI parent OR AB parent) OR (TI parents OR AB parents) OR (TI parental OR AB parental) OR (TI mother OR AB mother) OR (TI mothers OR AB mothers) OR (TI father OR AB father) OR (TI fathers OR AB fathers) OR (TI carer OR AB carer) OR (TI carers OR AB carers) OR (TI caregiver OR AB caregiver) OR (TI caregivers OR AB caregivers))  AND  ((MH Emotions+) OR (TI concern OR AB concern) OR (TI concerns OR AB concerns) OR (TI worry OR AB worry) OR (TI worries OR AB worries) OR (TI wellbeing OR AB wellbeing) OR (TI well-being OR AB well-being) OR (TI "well being" OR AB "well being") OR (TI emotion OR AB emotion) OR (TI emotions OR AB emotions) OR (TI emotional OR AB emotional))  AND  ((MH "child development disorders, pervasive+") OR (TI ASD OR AB ASD) OR (TI autism OR AB autism) OR (TI "autism spectrum disorder" OR AB "autism spectrum disorder") OR (TI autistic OR AB autistic) OR (TI "pervasive developmental disorder" OR AB "pervasive developmental disorder") OR (TI "PDD- NOS" OR AB "PDD- NOS") OR (TI asperger OR AB asperger) OR (TI aspergers OR AB aspergers))  AND  ((MH diagnosis+) OR (TI Diagnosis OR AB Diagnosis) OR (TI diagnostic OR AB diagnostic) OR (TI diagnosed OR AB diagnosed) OR (TI screening OR AB screening) OR (TI risk OR AB risk) OR (TI risks OR AB risks)) |

**Supplementary File 2. Mixed Methods Appraisal Tool (MMAT), Version 2018 Criteria**

| **Category of Study** | **Methodological quality criteria** |
| --- | --- |
| **Screening questions (for all types)** | S1. Are there clear research questions? |
|  | S2. Do the collected data allow to address the research questions? |
|  | *Further appraisal may not be feasible or appropriate when the answer is ‘No’ or ‘Can’t tell’ to one or both screening questions.* |
| **1. Qualitative** | 1.1. Is the qualitative approach appropriate to answer the research questions? |
|  | 1.2 Are the qualitative data collection methods adequate to address the research question? |
|  | 1.3. Are the findings adequately derived from the data? |
|  | 1.4 Is the interpretation of results sufficiently substantiated by data? |
|  | 1.5 Is there coherence between qualitative data sources, collection, analysis and interpretation? |
| **2. Quantitative randomised controlled trials** | 2.1. Is randomisation appropriately performed? |
|  | 2.2. Are the groups comparable at baseline? |
|  | 2.3. Are there complete outcome data? |
|  | 2.4. Are outcome assessors blinded to the intervention provided? |
|  | 2.5. Did the participants adhere to the assigned intervention? |
| **3. Quantitative non-randomised** | 3.1. Are the participants representative of the target population? |
|  | 3.2. Are measurements appropriate regarding both the outcome and intervention (or exposure)? |
|  | 3.3. Are there complete outcome data? |
|  | 3.4. Are the confounders accounted for in the design and analysis? |
|  | 3.5. During the study period, is the intervention administered (or exposure occurred) as intended? |
| **4. Quantitative descriptive** | 4.1. Is the sampling strategy relevant to address the research question? |
|  | 4.2. Is the sample representative of the target population? |
|  | 4.3. Are the measurements appropriate? |
|  | 4.4. Is the risk of nonresponse bias low? |
|  | 4.5. Is the statistical analysis appropriate to answer the research question? |
| **5. Mixed methods** | 5.1. Is there an adequate rationale for using a mixed methods design to address the research question? |
|  | 5.2. Are the different components of the study effectively integrated to answer the research question? |
|  | 5.3. Are the outputs of the integration of qualitative and quantitative components adequately interpreted? |
|  | 5.4. Are divergences and inconsistencies between quantitative and qualitative results adequately addressed? |
|  | 5.5. Do the different components of the study adhere to the quality criteria of each tradition of the methods involved? |

*From:* Hong, Q. N., Fàbregues, S., Bartlett, G., Boardman, F., Cargo, M., Dagenais, P., Gagnon, M. P., Griffiths, F., Nicolau, B., O’Cathain, A., Rousseau, M.-C., Vedel, I., & Pluye, P. (2018). The Mixed Methods Appraisal Tool (MMAT) version 2018 for information professionals and researchers. *Education for Information*, *34*(4), 285-291. <https://doi.org/10.3233/EFI-180221>

**Supplementary File 3. PRISMA 2020 Flow Diagram**

**Identification of studies via databases and registers**

Records removed *before screening*:

Duplicate records removed (n = 2940)

Records identified from*:

Databases (n = **6835**)

References: Forwards + Backwards (n = 752)

**Identification**

Records screened

(n = 4647)

Records excluded**

(n = 4576)

Reports sought for retrieval

Search (n=54)

F+B (n=17)

Reports not retrieved

(n = 0)

**Screening**

Reports excluded:

ASD screening/checklist (n = 16)

General parent concerns (n = 12)

Initial concerns or concerns overtime (n = 8)

Previous diagnosis (n = 5)

Concerns not categorised following diagnostic criteria (n = 5)

Retrospective (n = 4)

Data not presented as N (%) (n = 4)

Qualitative (n = 1)

Same sample as other included study (n = 3)

Diagnostic experiences (n = 1)

Talk/presentation (n = 1)

Treatment (n = 1)

Reports assessed for eligibility

(n = 71)

Studies included in review

(n = 10)

**Included**

*From:*  Page MJ, McKenzie JE, Bossuyt PM, Boutron I, Hoffmann TC, Mulrow CD, et al. The PRISMA 2020 statement: an updated guideline for reporting systematic reviews. BMJ 2021;372:n71. doi: 10.1136/bmj.n71

For more information, visit: <http://www.prisma-statement.org/>

**Supplementary File 4. Additional Study Characteristics**

| **Study** | **Study Aim** | **Inclusion Criteria** | **Exclusion Criteria** | **Interventions/ Programs Prior to Assessment** | **Parental Education** | **Socioeconomic Status** | **Diagnostic Criteria** | **Measurement Tool** | **Coding Technique**  **(And process)** |
| --- | --- | --- | --- | --- | --- | --- | --- | --- | --- |
| Azad et al., (2021), *United States of America* | To examine racial differences in parental beliefs or concerns and clinical judgment using convergent mixed-method approach. | - Parents agreed to join the approved research registry. - No previous evaluation for diagnosis of ASD (except in education settings). - Confirmed diagnosis of ASD using Autism Diagnostic Observation Schedule and a team-based clinical judgement. - Parent concerns collected before diagnostic evaluation. | Not reported. | - Children receiving intervention (61%). | - High school (25%). - Trade school (16%). - Bachelor (28%). - Graduate (31%). | - Underserved children.   **By study definition.* | - DSM-IV - DSM-5 | **Intake questionnaire:**  Questions asked:  a) Regardless of clinician opinion (such as previous doctors), do you believe that your child has ASD?  B) What are your concerns about your child? | **Codebook:**   - 60 codes comprised of 12 themes: - Academic - Cognition - Developmental - Emotional/behavioural - Life skills - Seeking diagnostic clarity or resources - Motor skills - Atypical behaviours - Health - Sensory - Social - Communication - Two ASD specific themes to assess whether ASD severity was associated with ASD specific parent concerns: - Restricted and repetitive behaviours; and - Communication.   **This process has been used in previous clinic papers (Pfeiffer et al., 2021).*  **Process:**   - Two independent coders. |
| Coffield et al., (2020), *United States of America* | To examine differences in the number and type of concerns among parents of underserved young children at risk of ASD. | - Children referred for evaluation due to developmental concerns raised by health care providers or parents, and thus considered high-risk for ASD. - Insured by Medicaid or uninsured; and/or non-White and/or Latino and/or spoke primary languages other than English. | Not reported. | Not reported. | - High school or less (49%). - Post high school education (44%). - Not reported (7%). | - Underserved children.   **By study definition.*   - Medicaid insured (95%). | - DSM-5 | **Developmental Check In Forms (Janvier et al., 2019):**   - Open-ended question regarding concerns about child’s development or behaviour.   **Structured Parent Interview:**   - To gather child and family health information, administration of the Mullen Scales of Early Learning and administration the Autism Diagnostic Observation Schedule 2^nd^ Edition. - Conducted by a developmental paediatrician, advanced practice nurse or licensed psychologist. | **Ozonoff et al., (2009) Coding Scheme:**  *Adapted version (Richards et al., 2016; Donahue et al., 2019).*   - ASD specific concerns: - Speech/language/ communication - Social - Stereotyped behaviour - Unspecified ASD - Non-ASD specific concerns: - Motor - Medical - Behaviour/temperament - General development   *Comments in Spanish were translated into English by a certified bilingual translator.*  **Process:**   - Two authors transcribed and coded. - Authors were blind to ASD diagnosis and demographic characteristics. - Discrepancies were resolved through consensus of five authors. |
| Coonrod and Stone, (2004), *United States of America* | To examine similarities and differences in parental report of early concerns about development in matched groups of 2-year-old children with and without autism. | - A diagnostic evaluation prior to age 3. - Availability of cognitive test scores. - Absence of identified metabolic or genetic disorders. - Absence of severe sensory and motor impairments. | Not reported. | Not reported. | - High school or beyond (93%). | Not reported. | - DSM-III - DSM-IV | **Early Concerns Questionnaire:**  *Open ended questionnaire developed for this study:*   - Age of first concerns: - "How old was your child when you first thought there might be something unusual about his/her behaviour or development?" - Nature of first concerns: - "What did you think was unusual?" - Age first sought help: - "How old was your child when you first tried to get professional help, or tried to find out what was wrong?" - Nature of current concerns: - "What do you see as the main problem now?" | **Codebook:**  Coded into six codes that aligned with Autism Diagnostic Interview-Revised:  1) Delayed or deviant language development (e.g., poor receptive or expressive language skills, no responsiveness to sounds).  2) Medical problems or delay in developmental milestones other than language (e.g., motor delays, general cognitive delays).  3) Lack of social interest or abnormality in social or emotional responses (e.g., poor eye contact, social disinterest/difficulty engaging or not wanting to be held).  4) Behaviour problems not specific to autism (e.g., sleeping problems, inattention, aggressive behaviour).  5) Autistic-type behaviours (e.g., motor stereotypes, repetitive behaviours, unusual play with objects).  6) Coding unclear or missing responses.  **Process:**   - Coded by two examiners. - Coders blind to diagnostic status. - Calculated percentage agreement and Cohen's kappa for initial and current concerns in each code. - High reliability of 95%-100% per category. |
| Herlihy et al., (2015), *United States of America* | To compare type and age of parent's first concerns in toddlers with autism spectrum disorder, prior to diagnosis between parents with and without older children with ASD. | - Selected from an ongoing study. - Screened positive on the Modified Checklist for Autism in Toddlers or Checklist for Autism in Toddlers, Revised. - Received an ASD diagnosis at the time of evaluation but having no prior diagnosis. - Having data about the presence or absence of siblings. - Completed responses to items of interest on the History Form or Autism Diagnostic Interview-Revised. | Not reported. | Not reported. | *Maternal education in years (Mean (SD)):*   - Siblings with ASD: 15.1 (2.7). - Typically developing siblings: 13.9 (2.6). - No siblings: 14.5 (2.1). | Not reported. | - DSM-IV-TR | **History Form:**   - Open-ended and multiple choice related to family composition, pregnancy and birth history, developmental milestones, and family health history. - Coded question: - "Please describe what worries you most about the child." | **Coded:**   - ASD specific concerns: - Communication/language concerns - Social deficits - Restricted or repetitive behaviours - Confirmed or suspected ASD - Non-ASD specific concerns: - Motor difficulties - Inattention and hyperactivity - Cognitive and developmental delays - Skills regression - Eating/feeding - Sensory difficulties - Sleep - Referral from a doctor or treatment provider - Tantrums or inappropriate behaviours - Others   **Process:**   - All concerns were coded without reference to the order in which they were listed. |
| Hess and Landa (2012), *United States of America* | To examine how parent social and communication concerns relate to similar domains on the Autism Diagnostic Observation Schedule and Mullen Scales of Early Learning. | - Younger siblings of a proband with idiopathic autism. - Part of a federally funded research project focused on understanding early markers for, and developmental trajectory associated with ASD. - Parent concerns collected prior to the start of the child's evaluation and prior to any feedback given by the clinician regarding the child's performance. | - Primary language exposure other than English. - Birth weight <2,500g. - Gestational age <35 weeks. - Severe birth trauma. - Head injury. - Prenatal illicit drug or excessive alcohol exposure. - Known genetic disorder that would confer increased risk for autism (e.g., fragile X). - Severe defects. | Not reported. | Not reported. | - Socioeconomic status ranged from 30-66 (M=57.36, SD=8.97) with higher scores indicating higher socioeconomic status. | Not reported. | **Three Questionnaires:**  1) Asked parents about present concerns. Permissible responses were 'Yes' or 'No'. If 'Yes' parents were requested to list their concerns.  2) Communication and Symbolic Behaviour Scale Developmental Profile-Caregiver Questionnaire (Wetherby and Prizant, 2002), with open ended questions regarding children's:   - 1. Communication changes   2. Strengths/assets   3. Concerns about development   4. Anything else regarding the child's development that parents wished to share.   3) The Sensory Profile (Dunn, 1999):   - Two open-ended questions regarding children's strengths and parental concerns. | **Codebook:**  Responses categorised into the following domains:   - Communication (including speech, language, communication, and social communication). - Motor (including fine and gross). - Social. - Behaviour/temperament (including stereotyped/repetitive behaviours and interests; challenging behaviours such as aggression or negativity; temperamental characteristics such as strong-willed or stubborn). - Sensory (including sensory defensiveness or sensory seeking). - Other (including anxiety, adaptive skills, somatic or medical issues etc.). - 'No Response".   **Process:**   - Coders blinded to diagnostic status. - Concerns were tallied and categorised with no attempt to prioritise concerns. |
| Jacobs et al., (2020), *Belgium*  Jacobs et al., (2020), *Belgium* | To investigate parents’ views and experiences of the ASD diagnosis before, right after and 12 months after their child was diagnosed. | - Flemish parents of young children (up to 6 years of age). - Children recruited prospectively, and it was unknown if they would get an ASD diagnosis. | - Previously diagnosed disability (Intellectually Disabled or other disability) who applied for a diagnostic ASD assessment. | Not reported. | Parents’ education and employment were diverse. | Not reported. | - DSM | **Three consecutive interviews:**  *T1 - Pre-diagnosis interviews:*   - Semi-structured by topic guide - Topics concerned the parents’: - Understanding of an ASD diagnosis. - Expectations regarding the implications of their child’s ASD diagnosis about treatment. - Psychological and relational experiences.   *T2 - After the diagnosis interviews:*   - Phenomenological in-depth interviews. - Conducted within 2-weeks after parents’ feedback session at the end of their child’s assessment.   *T3 - Post-diagnosis interviews:*   - Phenomenological in-depth interviews. - Conducted 12-months after the T2 interview. | **Process:**   - Data analysed by applying to procedures outlined for interpretative phenomenological analysis. - Recorded interviews transcribed. - Systematically and inductively coded transcripts in NVivo 11. - Two reviewers combined relevant codes across interviews to discern patterns. - All four authors met regularly to discuss and govern this analytic process in an interdisciplinary collaboration. - Authors translated representative quotations verbatim from Dutch to English to give an impression of interviewees’ original expressions. |
| Jayanath and Ozonoff, (2020), *Malaysia* | This study aims to:  a) determine the age of first parental concern and the type of parental concern for paediatric patients who subsequently received a diagnosis of ASD  b) determine the association between first parental concern(s) and ASD severity  c) ascertain the time lag between the age of first parental concern and a diagnosis  d) identify factors associated with a delayed diagnosis (age >36 months). | - All patients had a documented diagnostic confirmation of ASD by a developmental paediatrician. | - Patients younger than one year and older than 18 years were excluded | Not reported. | Not reported. | Not reported. | - DSM-5 | **Electronic Medical Records:**   - Review of Electronic Medical Records from of doctors at the clinic. | **Ozonoff et al., (2009) Coding Scheme:**  Coded into one of 10 categories:   1. No concerns 2. Speech/language/ communication concerns 3. Social behaviour concerns 4. Stereotyped behaviour concerns 5. Motor concerns 6. Medical concerns 7. Unspecified concerns related to autism 8. General developmental concerns 9. Other concerns   **Process:**   - One medical graduate familiar with the clinics charting procedures and Electronic Medical Record systems extracted all information. - Review of the quality and consistency of all data collected was done daily by the supervisor. - Reliability between coders was achieved at 97.5% for 20% of concerns. |
| Richards et al., (2016), *United States of America* | To examine the relationship between concerns parents reported and the diagnosis toddlers received in a sample of children who screened positive on an ASD screening questionnaire. | - Participant data extracted from two studies (Chlebowski et al., 2013; Robins et al., 2014), validating the Checklist for Autism in Toddlers and its current version Checklist for Autism in Toddlers, Revised. - Toddlers were required to be screened during well-child check-ups. | - Children had an ASD diagnosis prior to screening. - Sensory or motor disabilities prevented them from completing the standardised assessment measures. - Child's caregiver was not fluent in English. - History questionnaire did not contain information on whether a child received a diagnosis in between screening and diagnostic evaluation. | - Received any type of therapy (63%). - Saw Any type of specialist 49%). | 4-year college degree (29%):   - Bachelors (18%). - Graduate degree (12%).   Less than 4-year degree (50%):   - No degree or diploma (11%). - High school diploma/ general educational development (22%). - Associates degree or technical degree (13%). | Not reported. | - DSM-IV | **History Questionnaire:**  Three items were coded from the questionnaire:   - "Please briefly list any concerns/worries you have about how the child has been developing (walking, speaking, playing) or behaving." - "Concerns about your child's development." - "What are the first things that made you concerned about your child's development? How old was your child at that time?" | **Ozonoff et al., (2009) Coding Scheme:**  The current studies concern categories differed from those in the original Ozonoff et al., (2009) version in three ways:   - 1. Separated out feeding/eating concerns from other medical/regulatory concerns.   2. Included sensory concerns with stereotyped behaviour concerns.   3. Combined general concerns with development concern.   *Coded into four ASD-related concerns:*   1. Speech/communication 2. Restricted/repetitive behaviours (including sensory) 3. Social skills 4. Named ASD, (i.e., parents named autism/ASD specifically)   *Coded into five Non-ASD specific concerns:*   1. Motor 2. Behavioural/temperament 3. Medical/regulatory 4. Eating/feeding 5. Unspecified   **Process:**   - Preliminary analyses involved evaluation of raw and coded frequency distributions to determine the statistical characteristics of the data. |
| Wallisch et al., (2020), *United States of America* | To examine the extent to which specific parent concerns differentiate multiple diagnostic categories in comparison to children with ASD among a large university medical centre sample of children ages 3-6 years. | - Children were between 36-72 months of age. - Parents had completed intake paperwork (before diagnosis). - A diagnostic decision was documented in their record. | Not reported. | Not reported. | Not reported. | Not reported. | - DSM-IV - DSM-V | **Intake paperwork:**   - Open ended question response to listing three top concerns at that point in time. | **Ozonoff et al., (2009) Coding Scheme:**  Adapted version that included the following categories:  1) Behaviour/temperament  2) Motor  3) General development  4) Speech/communication  5) Social interactions  6) Stereotyped behaviour  7) Medical/medication questions  8) Sensory aversions/preferences  **Process:**   - Two researchers coded. - Coded agreement was tested on 20% of the dataset, and 89% agreement was achieved. |

*There is a partial participant overlap between Herlihy et al., (2015) and Richards et al., (2016).*

*ASD = Autism Spectrum Disorder; DSM-IV = Diagnostic Manual of Mental Disorders, fourth edition; DSM-5 =Diagnostic Manual of Mental Disorders, fifth edition; DSM-III = Diagnostic Manual of Mental Disorders, third edition; DSM-IV-TR = Diagnostic Manual of Mental Disorders, fourth edition, text revision.*

**Supplementary File 5. Data Extraction**

| **Study** | **Sample**  **Number (N)** | **ASD Specific Concerns** | | | | | | **Non-ASD Specific Concerns** | | | | | | | | | |
| --- | --- | --- | --- | --- | --- | --- | --- | --- | --- | --- | --- | --- | --- | --- | --- | --- | --- |
|  |  | **Speech/ Language/ Communication** | | **Social** | | **Stereotyped Behaviours** | | **Behaviour/ Temperament/ Emotional** | | **Developmental/ General** | | **Medical/ Regulatory** | | **Sensory Aversions/ Preferences** | | **Motor** | |
|  |  | **N** | **%** | **N** | **%** | **N** | **%** | **N** | **%** | **N** | **%** | **N** | **%** | **N** | **%** | **N** | **%** |
| Azad et al., (2021), United States of America | 489 children | 298 | 60.9 | 249 | 50.9 | 83 | 17.0 | 193 | 39.5 | 44 | 9.0 | 55 | 11.2 | 72 | 14.7 | 37 | 7.6 |
| Coffield et al., (2020), United States of America | 288 children | 149 | 51.7 | 55 | 19.1 | 47 | 16.3 | 109 | 37.8 | 25 | 8.7 | 21 | 7.3 |  |  | 10 | 3.5 |
| Coonrod and Stone, (2004), United States of America | 44 children | 37 | 84.1 | 3 | 6.8 |  |  | 6 | 13.6 |  |  |  |  |  |  |  |  |
| Herlihy et al., (2015), United States of America | 69 children | 38 | 55.1 | 14 | 20.3 | 4 | 5.8 |  |  |  |  |  |  |  |  |  |  |
| Hess and Landa (2012), United States of America | 63 parents | 17 | 27.0 | 13 | 20.6 |  |  | 13 | 20.6 |  |  |  |  | 5 | 7.9 | 5 | 7.9 |
| Jayanath and Ozonoff, (2020), Malaysia | 366 children | 299 | 81.7 | 221 | 60.4 | 57 | 15.6 | 83 | 22.7 | 36 | 9.8 | 13 | 3.6 |  |  | 12 | 3.3 |
| Richards et al., (2016), United States of America | 532 children | 418 | 78.6 | 123 | 23.1 | 63 | 11.8 | 140 | 26.3 | 61 | 11.5 | 88 | 16.5 |  |  | 164 | 30.8 |
| Wallisch et al., (2020), United States of America | 503 children | 218 | 43.3 | 134 | 26.6 | 61 | 12.1 | 194 | 38.6 | 115 | 22.9 | 102 | 20.3 | 33 | 6.6 | 36 | 7.2 |

**Supplementary File 6. MMAT Results**

| **Study** | **Study Type**  **(As defined by study)** | **Screening Questions** | | **Qualitative** | | | | | **Quantitative Descriptive** | | | | | **Mixed Methods** | | | | | **MMAT Notes** |
| --- | --- | --- | --- | --- | --- | --- | --- | --- | --- | --- | --- | --- | --- | --- | --- | --- | --- | --- | --- |
|  |  | **S1** | **S2** | **1.1** | **1.2** | **1.3** | **1.4** | **1.5** | **4.1** | **4.2** | **4.3** | **4.4** | **4.5** | **5.1** | **5.2** | **5.3** | **5.4** | **5.5** |  |
| Azad et al., (2021), United States of America | Mixed Methods | Y | Y | Y | Y | Y | Y | Y | Y | N | Y | Y | Y | Y | Y | Y | Y | Y | 4.2 Participants not representative of the general population. |
| Coffield et al., (2020), United States of America | Qualitative/Mixed Methods | Y | Y | Y | Y | Y | Y | Y | Y | Y | Y | Y | Y | N | Y | Y | C/T | Y | 5.1 Does not justify why both methods were used.  5.4 Unclear on divergences reported. |
| Coonrod and Stone, (2004), United States of America | Quantitative Descriptive | Y | Y |  |  |  |  |  | Y | N | Y | C/T | Y |  |  |  |  |  | 4.2 Participants not representative of the general population.  4.4 Does not explain how excluded children may have impacted results. |
| Herlihy et al., (2015), United States of America | Quantitative Descriptive | Y | Y |  |  |  |  |  | Y | Y | Y | C/T | Y |  |  |  |  |  | 4.4 No descriptions on how many participants could have possibly been included. |
| Hess and Landa (2012), United States of America | Quantitative Descriptive | Y | Y |  |  |  |  |  | Y | Y | Y | N | Y |  |  |  |  |  | 4.4 Cohort study and unclear how many other participants were excluded and why. |
| Jacobs et al., (2020), Belgium  Jacobs et al., (2020), Belgium | Qualitative | Y | Y | Y | Y | Y | Y | Y |  |  |  |  |  |  |  |  |  |  | Met all criteria |
| Jayanath and Ozonoff, (2020), Malaysia | Quantitative Descriptive | Y | Y |  |  |  |  |  | Y | N | Y | C/T | Y |  |  |  |  |  | 4.2 Participants not representative of the general population.  4.4 Does not explain how many participants were excluded and why. |
| Richards et al., (2016), United States of America | Quantitative Descriptive | Y | Y |  |  |  |  |  | Y | Y | Y | C/T | Y |  |  |  |  |  | 4.4 Unclear if non-response bias was reported. |
| Wallisch et al., (2020), United States of America | Quantitative Descriptive | Y | Y |  |  |  |  |  | Y | N | Y | C/T | Y |  |  |  |  |  | 4.2 Participants not representative of the general population.  4.4 Does not disclose how many children could have participated and why. |

***Key:*** *Green Y = Yes; Red N = No; Yellow C/T = Can’t Tell; and blank squares means criteria was not applicable.*
